# Softness of hydrated salt crystals under deliquescence

**DOI:** 10.1038/s41467-023-36834-0

**Published:** 2023-02-25

**Authors:** Rozeline Wijnhorst, Menno Demmenie, Etienne Jambon-Puillet, Freek Ariese, Daniel Bonn, Noushine Shahidzadeh

**Affiliations:** 1 Institute of Physics, Van der Waals-Zeeman Institute, University of Amsterdam, Science Park 904, 1098 XH Amsterdam, The Netherlands; 2 LaserLaB, Biophotonics and Medical Imaging, Vrije Universiteit Amsterdam, De Boelelaan 1081, 1081 HV Amsterdam, The Netherlands; 3 Present Address: Laboratory for Soft and Living Materials, Department of Materials, ETH Zurich, Vladimir-Prelog-Weg 1-5, Zurich, 8093 Switzerland

**Keywords:** Fluids, Chemical physics

## Abstract

Deliquescence is a first-order phase transition, happening when a salt absorbs water vapor. This has a major impact on the stability of crystalline powders that are important for example in pharmacology, food science and for our environment and climate. Here we show that during deliquescence, the abundant salt sodium sulfate decahydrate, mirabilite (Na_2_SO_4_·10H_2_O), behaves differently than anhydrous salts. Using various microscopy techniques combined with Raman spectroscopy, we show that mirabilite crystals not only lose their facets but also become soft and deformable. As a result, microcrystals of mirabilite simultaneously behave crystalline-like in the core bulk and liquid-like at the surface. Defects at the surface can heal at a speed much faster than the deliquescence rate by the mechanism of visco-capillary flow over the surface. While magnesium sulfate hexahydrate (MgSO_4_⋅6H_2_O) behaves similarly during deliquescence, a soft and deformable state is completely absent for the anhydrous salts sodium chloride (NaCl) and sodium sulfate thenardite (Na_2_SO_4_). The results highlight the effect of crystalline water, and its mobility in the crystalline structure on the observed softness during deliquescence. Controlled hydrated salts have potential applications such as thermal energy storage, where the key parameter is relative humidity rather than temperature.

## Abstract (web summary)

Crystalline solids are commonly associated with their hard and faceted nature. Here, the authors report the transition from hard to soft and deformable, observed in the gradual dissolution of salt crystals that harbor water in their crystalline framework.

## Introduction

Minerals are natural materials that make up a large part of the Earth and other planets^1,2^. Their physical properties are generally determined by their constituent chemical elements, the crystal lattice defining the arrangement of atoms and the nature of bonding. Properties of minerals such as fluorescence, magnetism or radioactivity are of great technological significance. Among these properties is the crystal’s tendency to absorb moisture and be dissolved by water, which is called deliquescence and occurs above a critical relative humidity, also known as the deliquescence point^3–6^. This phenomenon is important to a wide range of applications such as chemical etching or the stability of pharmaceutical formulations, stability of food ingredients^7^ and in Earth and atmospheric science^8,9^. In the latter field, aerosol absorption by minerals has large impact on climate and environment. Inorganic salts due to their ionic structure and the solubility of ions in water are in principle hygroscopic. They gradually absorb moisture from the atmosphere in such a way that they dissolve very slowly by forming a saturated salt solution.

Although the solid stays in a crystalline state during dissolution, a decrease in the overall crystal roughness and a smoothening of crystal edges and corners are observed^10,11^. This process of changing crystal shape and properties due to deliquescence is known to be dependent on the level of undersaturation of the atmosphere to which the crystal is exposed^12^. Although extensive research has been performed for dissolution at high undersaturation (far from equilibrium and in pure solvent), much less is known about the microscale effects of dissolution at low undersaturation.

Here we describe the behavior of hydrated sodium sulfate crystals at their deliquescence point compared to anhydrous salts. Hydrates are inorganic salts which contain water molecules in a definite ratio as an integral part of the crystalline structure. The water is stoichiometrically bound into a crystal but not directly bonded to the central metal cation. We show by combining direct microscopy observations with Raman spectroscopy that above the deliquescence point, microcrystals of natural inorganic sodium sulfate decahydrate, known as mirabilite, behave differently than anhydrous crystals like thenardite and halite. Our results show that mirabilite crystals not only lose their facets but become soft and deformable at their surface; defects at the crystalline surface spontaneously and rapidly close themselves under the action of surface tension and viscous flow at a time scale much shorter than the deliquescence rate. We show that such behavior is completely absent during the deliquescence of thenardite and halite microcrystals. Despite some smoothening of the corners, the latter keep their faceted shape during the whole deliquescence process. The peculiar behavior of mirabilite appears to be related to the crystalline water and its mobility in the crystalline structure resulting in a gradual disordering at the surface region and conferring softness to the crystal during the slow process of deliquescence.

## Results

### Deliquescence of anhydrous Na_2_SO_4_ powder

**Fig. 1:**
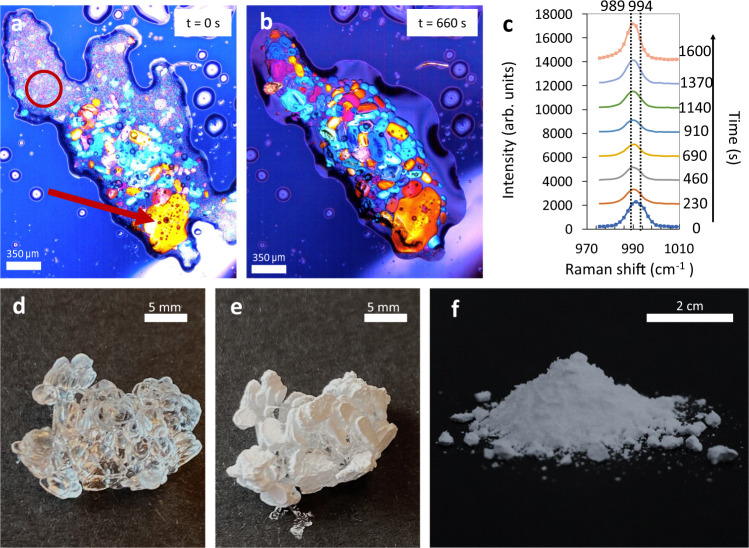
Gradual deliquescence of thenardite (Na_2_SO_4_) polycrystals and rapid precipitation of mirabilite (Na_2_SO_4_·10H_2_O) microcrystals. **a, b** Microscopy images made with cross polarizers of the deliquescence process of the dehydrated powder. The colors that are seen with cross-polarized light highlight the fact that what we are seeing is not a liquid structure. **a** Initially, both polymorphs (mirabilite and thenardite) coexist, as shown by the Raman spectra of the region circled in red. The red arrow shows entrapped remaining thenardite nanocrystals (black dots) in the mirabilite crystals. This has been confirmed by Raman spectroscopy of the ‘black spots’ and the crystal surrounding these spots, and subtracting them (see Supplementary Fig. 3). **b** After 660 s, most of the thenardite nanocrystals are dissolved; further water vapor absorption will cause the mirabilite crystals to deliquesce in turn. **c** Raman spectra of the evolution of the SO_4_ symmetric stretch peak during deliquescence of thenardite powder; the dotted lines indicate the peak maxima of thenardite (994 cm^−1^) and mirabilite (989 cm^−1^) (see Supplementary Figs. 2 and 8). **d** Typical mirabilite crystals precipitated out of a sodium sulfate solution (see Methods) **e** Same Mirabilite crystals dried (dehydrated) at *T* = 21 °C **f** Powder of **e** referred as thenardite (Na_2_SO_4_) polycrystals powder (see Supplementary Fig. 1) used for the deliquescence experiment.

Large numbers of microscale mirabilite crystals are formed by placing a small amount of the thenardite powder (see Methods and Fig. 1d–f) under a polarizing light microscope in a climate chamber at a relative humidity above the equilibrium relative humidity RH_eq_ ≈ 96% of this salt (Supplementary Fig. 2)^13^. First, the progressive moisture sorption and deliquescence of the thenardite nanocrystals result in the gradual formation of saturated salt solution with respect to thenardite. The solution being highly supersaturated (S ≈ *m*_thenardite_/m_mirabilite_ ≈ 2.5 at 21 °C) with respect to mirabilite (Supplementary Fig. 2), induces the precipitation of a large amount of mirabilite microcrystals (Fig. 1a, b). Raman identification of these crystals under confocal Raman microspectroscopy reveals first an overlay of the thenardite (994 cm^−1^) and mirabilite (989 cm^−1^) spectra; the intensity of the thenardite decreases gradually in time whereas that of the mirabilite spectrum increases with the formation and growth of more mirabilite microcrystals (Fig. 1c). In addition, as can be seen in Fig. 1a, b, under the cross-polarized microscope, each single crystal of mirabilite reflects its own color due to its configuration. The growth of mirabilite crystals proceeds until a certain size as the surrounding salt solution concentration drops to the saturation concentration in equilibrium with mirabilite crystals (Supplementary Fig. 2). Beyond this threshold, the mirabilite microcrystals in turn start to dissolve as the deliquescence process continues.

**Fig. 2:**
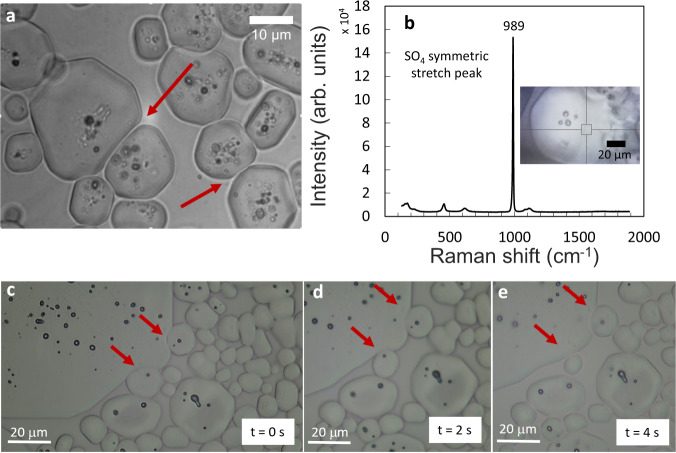
Assembly of mirabilite microcrystals above the deliquescence point. **a** Non-faceted mirabilite microcrystals are deformable and adapt their shape to neighboring crystals in the sodium sulfate solution. **b** Raman spectrum of the crystals identified as mirabilite. **c**–**e** The red arrows point to local deformation of a large mirabilite crystal, which is reversed once the strain is removed.

During deliquescence, the microcrystals not only lose their facetted shape and appear rounded but that they also become soft at the surface; this can be most clearly observed in regions with a high local density of crystals: the microcrystals are observed to deform each other and adapt to the shape of neighboring crystals (Fig. 2a, Supplementary Movie 1). Once the strain imposed by the presence of other crystals is removed, the deformed crystals regain their non-faceted spherical shape (Fig. 2c–e). Taking the Raman spectrum of such a deformable crystal reveals a mirabilite spectrum and confirms the observations during deliquescence (Fig. 2b). When removing the climatic chamber and thus decreasing the relative humidity below the equilibrium relative humidity, the crystals regain their facets (Supplementary Fig. 7).

### Self-healing behavior at the crystal surface

**Fig. 3:**
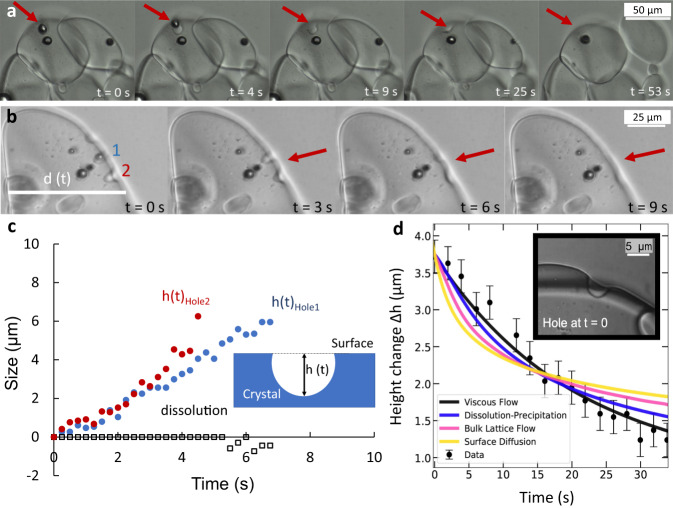
Self-healing behavior at the surface of mirabilite crystals. **a, b** Evolution of surface defects on two different crystals, indicated by red arrows. During deliquescence, while the entrapped thenardite dissolves, the indentation at the surface of the hydrated crystal is seen to level out rapidly. **c** Levelling out *Δh(t) = h(t) − h*_0_ of two holes (filled circles, hole 1 in blue, hole 2 in red) and dissolution *Δd(t) = d(t) − d*_0_ of the same crystal (open squares). **d** Comparison between the measured depth evolution of a hole *h*(*t*) (symbols) and the Mullins model (solid lines) describing the four possible mechanisms to explain the levelling out (black viscous flow, blue dissolution-precipitation, magenta bulk lattice flow, yellow surface diffusion, black dots raw data, the error bars are based on the pixel size of the raw data). The model describing a viscous flow (black curve) shows the best agreement with the data.

In addition, we also observe in a separate set of experiments (Fig. 3, Supplementary Movie 1) that indentations, holes, or defects at the crystal surface level out spontaneously, thus flattening the surface.

The levelling out of the holes is not due to the overall deliquescence of the mirabilite crystal: comparison of the dissolution speed of the crystals *d(t)* with the temporal evolution of the depth of several holes *h(t)* of different sizes on various mirabilite crystals shows that the levelling out of the holes is much faster than the deliquescence rate (Fig. 3c). One way to interpret these results is to assume that the mobility of the molecules at the crystalline surface is very high. To determine the mechanism responsible for that, we use the Mullins model^14–16^ which solves the levelling out of a hole due to four possible mechanisms: (1) viscous-capillary flow of the crystal, (2) dissolution precipitation of the salt from the solution into the holes, (3) bulk lattice rearrangement, and (4) rearrangement of surface molecules by surface diffusion. We compare this model to the hole profiles measured during levelling out (Supplementary Fig. 5) and to the hole depth evolution (Fig. 3d) and find that the viscous-capillary flow mechanism is the only one able to describe the data (for details on the model, equation, and comparison with experiments, Methods).The excess capillary energy introduced by the curvature of the hole with respect to the flat equilibrium state is dissipated by viscous flow into the hole that leads to the observed levelling out similar to self-healing in polymers^16^ and curved colloidal crystals^17^. For the latter, the self-healing behavior has been interpreted as collective particle rearrangement with the underlying curvature. Here, the relaxation of the holes at the surface is thus driven by gradients in Laplace pressure along the crystal-solution interface, meaning that the driving force for the healing is the surface tension that requires the crystals to be spherical. A fitting parameter in the Mullins model is the capillary velocity; its value is found to be approximately 0.1 μm s^−1^ (Supplementary Table 1) for the hole shown in Fig. 3d and does not vary much between different experiments.

### Behavior of anhydrous salts during deliquescence

**Fig. 4:**
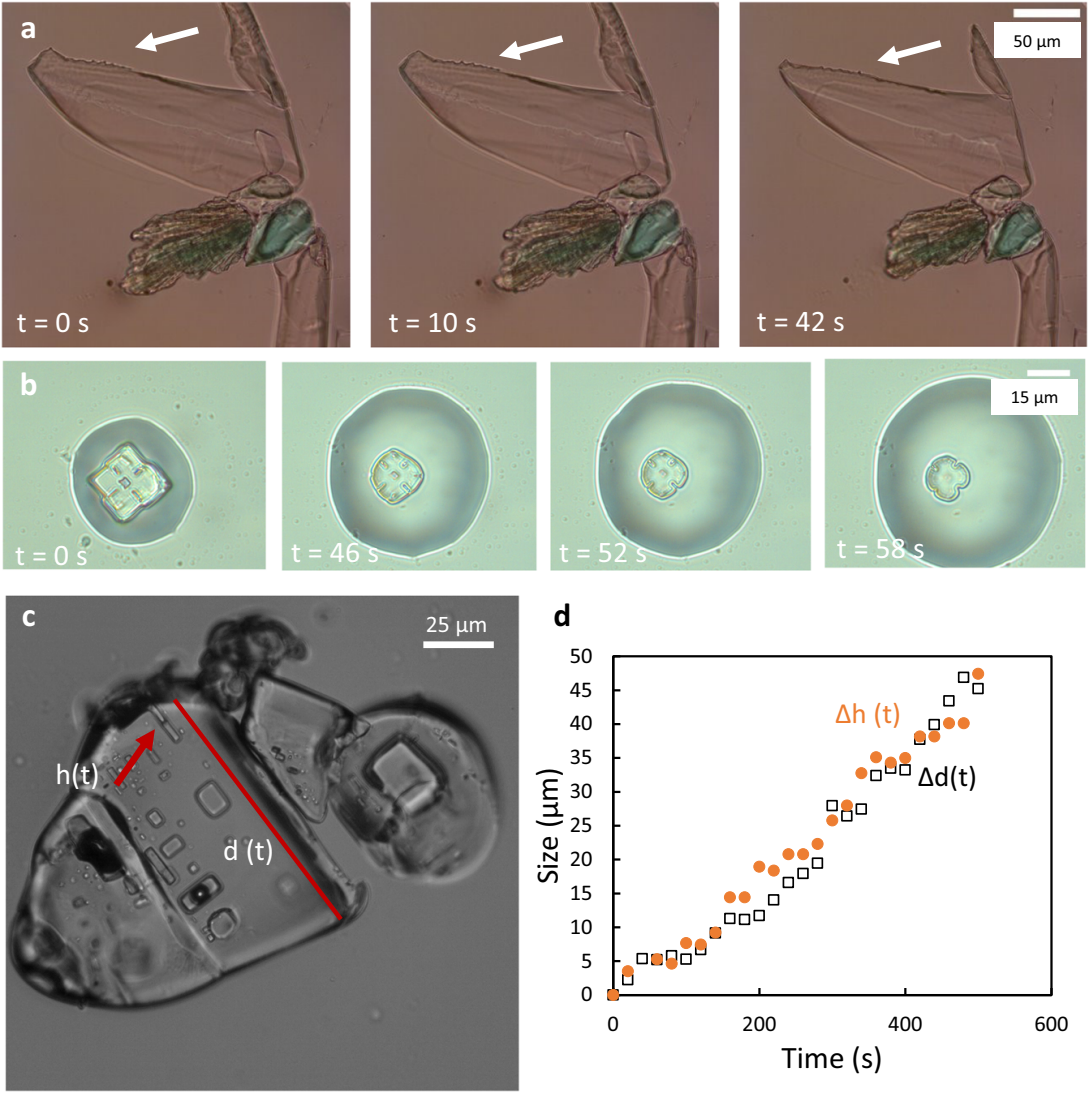
Deliquescence over time of anhydrous salts at RH ≈ 100%, *T* = 21 °C. **a** Thenardite (Na_2_SO_4_) crystal above the deliquescence RH ≈ 96%. Facets are visible and defects at the surface (white arrows) remain in place during deliquescence. **b** Sodium chloride crystal, above deliquescence RH ≈ 75%. Surface defects keep their shape while the corners smoothen. **c, d** The decrease of the crystal size *d(t)* and the height of the defect *h(t)* at the surface (red arrow) follow the same speed.

To check if this behavior is specific to the mirabilite crystal or rather a common behavior for different salts, the experiments are repeated for two anhydrous salts: thenardite (Na_2_SO_4_, the anhydrous form of sodium sulfate) and sodium chloride (Supplementary Movie 2), and for another hydrated crystal: magnesium sulfate hexahydrate (MgSO_4_·6H_2_O). In the latter case, similar non-faceted and soft microcrystals are observed under the microscope although a quantitative analysis is impossible with our setup and camera (see “Methods”, and Supplementary Fig. 6). In contrast, the anhydrous crystals of similar sizes do not show the same behavior during deliquescence: although their edges and corners become more rounded with dissolution, the microcrystals nevertheless maintain their faceted shape till complete deliquescence. In addition, defects at the surface of the crystals disappear with the same dissolution rate as the rest of the crystal (Fig. 4).

## Discussion

Hydrated crystals are common for salts that contain +2 and +3 cations as well as −2 anions. In some cases, like for mirabilite, the majority of the weight of a compound can arise from the crystalline water. This water is not covalently bonded to a host molecule or to ions. Mirabilite crystals have a layered structure in which chains of sodium octahedra are separated by sulfate tetrahedra which are connected through ordered and disordered hydrogen bonds of the interstitial water molecules^18^. The ease of exchange of water molecules in the crystalline structure can be studied by looking at how easily this water of hydration evaporates from a given crystalline structure. Generally, the higher the number of bounded water molecules in the crystal, the easier the dehydration^13,19,20^. For the sodium sulfate decahydrate, this readily happens at room temperature, whereas for hydrated salts with fewer water molecules in the lattice the water evaporates at higher temperatures. For example, magnesium sulfate hexahydrate should be heated above 60 °C for dehydration or for magnesium sulfate monohydrate and calcium sulfate dehydrate the temperature should be above 150 °C. For bivalent ions, the water can be more strongly bounded, again decreasing the exchange rate with the exterior water. The same reasoning can thus also explain why the softness at the crystal surface during deliquescence becomes less evident for crystals with less water in their structure (i.e., magnesium sulfate hexahydrate) and not observable in case of anhydrous salts.

Another important observation in this respect is that when the relative humidity is decreased below the equilibrium relative humidity RH_eq_ (i.e., below the deliquescence point), the unfaceted soft hydrated crystals become rigid and facets grow again over time (see Supplementary Fig. 7). Thus, this surface mobility only appears at the deliquescence point (i.e., above a certain RH for a given hydrated salt), reminiscent of the roughening transition that appears at a fixed temperature for certain crystals, and above which a solid core-liquid shell structure is reported. Temperature-induced shape changes at the roughening transition have been reported for nanocrystals crystals (such Au) smaller than 5 nm^21,22^. Here we are at larger scale, but our mirabilite microcrystals display a similar transition at isothermal conditions during deliquescence.

Our observations of the self-healing and softness of hydrated crystals are reminiscent of the case of Helium-4 crystals close to zero temperature, which under the application of small stresses present a plasticity due to the motion of crystalline defects. This leads, amongst other things, to the ‘dripping’ of such crystals, which are reported to behave liquid-like rather than solid-like due to the bulk lattice motion (mechanism (3) in our Mullins model)^16,23–25^. In our case of hydrated salts, the deformability of the microcrystals at the deliquescence point is mainly caused by capillary-driven viscous flow at the surface of the crystal. This governs the self-healing dynamics of the crystals and shows why the molecules in the crystalline lattice appear to be mobile, leading to spherical and deformable crystals on our experimental time scale. The time scale for the viscous flow limits the size range over which the deformability can be observed.

The hydrated salts that we study here are not only abundant and ubiquitous on the surface of the Earth but are also present on Mars^2,26,27^. In fact, their discovery on the sediments of Mars provided vital information on the hydrogeologic history of other planets. In addition, they are an important class of natural materials that are currently investigated both as thermochemical materials and phase change materials and for energy storage purposes because of their large potential storage capacity (≈1–3 GJ m^−3^) and the easily accessible temperature range that they cover^28,29^. Our results provide insights into the deliquescence, i.e., dissolution at low undersaturation, of hydrated salts compared to anhydrous crystals, which can help for optimizing their performance with improved control over their geometry and size. Such materials have potential for applications where the key parameter is relative humidity rather than temperature. More generally, the conception of soft microcrystalline materials with peculiar macroscopic properties with respect to their shape, color, and luminescence as ‘flexible responsive systems’ remains an underdeveloped area that deserves further exploration^30–32^.

## Methods

### Recrystallization of the anhydrous Na_2_SO_4_ nanocrystals in the form of powder

First, sodium sulfate solutions were prepared by dissolving anhydrous thenardite Na_2_SO_4_ (Sigma Aldrich; 99.8% purity) little by little while stirring in Millipore water (ρ ≈ 18.2 MΩ cm) up to a concentration of 18.6 wt % or 1.6 mol kg^−1^ of solvent; beyond this concentration the solution became turbid due to the precipitation of mirabilite crystals (Na_2_SO_4_·10H_2_O) from the supersaturated solution (concentration at saturation being *m*_s_ = 1.4 of solvent). The stirring was then stopped and the bottle kept at rest for one day at room temperature. Some precipitated mirabilite crystals were subsequently removed from the saturated salt solution and dried at ambient temperature and relative humidity RH∼45% of the lab. The stability of the hydrated/anhydrous phase in open air depends on the temperature and relative humidity. Upon drying and dehydration, the transparent mirabilite crystals became white and crumbly while keeping the shape of the initial hydrated crystals (see Fig. 1a, b). The composition and morphology of the white structure were identified by Raman spectroscopy and high-resolution scanning electron microscopy as being an assembly of thenardite nanocrystals.

We studied the formation of sodium sulfate decahydrate (Na_2_SO_4_·10H_2_O) by deliquescence of the anhydrous form of the salts using electron and optical microscopy as well as confocal Raman microspectroscopy. First, the anhydrous form of sodium sulfate was obtained by drying millimeter-sized mirabilite crystals precipitated in salt solution at room temperature. Mirabilite or sodium sulfate decahydrate, also referred to as Glauber’s salt^33^, is the thermodynamically stable state in contact with a saturated sodium sulfate solution at room temperature (Supplementary Information Fig. 1)^34^. However, once the crystals were taken out of solution, the water in the crystalline structure evaporated easily at room temperature, and subsequently the initially transparent hydrated crystal transformed into a white porous structure made up of micro crystallites, while retaining the overall shape of the original millimeter-sized mirabilite crystal (Fig. 1a, b). Scanning electron microscopy imaging showed that this dehydrated white porous structure was made of an assembly of microcrystals of thenardite (Fig. 1d), as identified by confocal Raman microscopy with an SO_4_^2-^symmetric stretch peak at 994 cm^−1^ (Supplementary Fig. 2) in agreement with previous work^35,36^. These porous structures were subsequently rehydrated by deliquescence.

### Deliquescence of anhydrous Na_2_SO_4_ nanocrystals

The anhydrous Na_2_SO_4_ powder was placed on a clean glass slide and compressed to minimize spaces between the powder particles. The glass slide was then placed into a controlled mini climatic chamber under the microscope and subjected to an increasing relative humidity. The physical changes of the powder due to moisture adsorption were followed under a Leica DM IRM inverted microscope equipped with a 800 × 600 pixels and 8-bit sensitivity CCD camera. The images of the ensuing process were taken at different time intervals at ×10, ×20, ×40, and ×60 magnifications. The images were subsequently analyzed using the Fiji ImageJ software.

### Recrystallization of the anhydrous MgSO_4_ nanocrystals in the form of powder

A similar procedure as for the recrystallization of the Na_2_SO_4_ was followed. First, magnesium sulfate solutions were prepared by dissolving anhydrous magnesium sulfate (Sigma Aldrich; 99.5% purity) little by little while stirring in Millipore water to obtain a supersaturated solution (concentration at saturation being *m*_s_ = 2.8 mol kg^−1^ of solvent). The stirring was then stopped and the bottle was kept at rest for one day at room temperature. Droplets of the solution were put on a Corning glass slide (borosilicate) and were dried at T = 60–65 °C.

After approx. 24 h the dried droplets were removed from the oven and crushed to obtain a white powder which was stored at RH = 20% to ensure the MgSO_4_ stayed anhydrous. The composition and the morphology of the powder was identified by Raman spectroscopy and high-resolution scanning electron microscopy as being anhydrous MgSO_4_ crystals.

### Deliquescence of anhydrous MgSO_4_ nanocrystals

The anhydrous MgSO_4_ powder was placed on a clean glass slide. The glass slide was then placed into a controlled mini climatic chamber under the microscope and subjected to an increasing relative humidity. The physical changes of the powder due to moisture adsorption were followed under a Leica DM IRM inverted microscope equipped with a 800 × 600 pixels and 8-bit sensitivity CCD camera. The images of the ensuing process were taken at different time intervals at ×10, ×20, ×40, and ×60 magnifications. The images were subsequently analyzed using ImageJ software. The deformable crystals appeared above RH ≈ 85%.

The process was, however, much faster than for the sodium salt since the deliquescence of magnesium sulfate starts at a lower equilibrium relative humidity RH_eq_ = 85%^37^, which induces a faster water vapor sorption rate in comparison with sodium sulfate in our climate chamber (where the relative humidity of the chamber is fixed at RH ≈ 100%). In addition, the magnesium sulfate hexahydrate microcrystals were smaller in size, and the indentations were too small and closed too rapidly to be able to measure quantitatively the dynamics with our camera and experimental setup.

### Confocal Raman spectroscopy

For chemical identification of elements and various polymorphs of Na_2_SO_4_ and MgSO_4_ during the deliquescence, we applied Raman microscopy using a Renishaw InVia spectrometer with a 532 nm laser calibrated with a silicon monocrystal. First, we took reference spectra in the fingerprint area of each compound: the mirabilite Na_2_SO_4_·10H_2_O, the anhydrous Na_2_SO_4_ powder obtained after dehydration, and the 1.4 mol kg^−1^ of solvent saturated solution, using ×20 and ×50 magnification objectives, backscattering geometry, and 100% laser power. These spectra helped to distinguish different phases as the wavenumber position and number of peaks differ for each phase. The peak values of the reference spectra were compared to the literature values and an excellent agreement was found^34^. We next took sequential Raman spectra to characterize the full deliquescence process and the transformation of different phases. The deliquescence was carried out in a similar mini climatic chamber as used with the inverted Leica microscope, but with an aluminum slide instead of a glass slide at the bottom. Furthermore, we also did single measurements on the hydrated crystals that appeared during deliquescence.

### Statistical test to determine the best fitting model for the levelling out of holes

To determine the driving mechanism behind the levelling out of the holes, we compared the data of the measurements with four candidate models: (1) relaxation by a viscous flow, (2) a diffusion process, (3) a bulk lattice flow, and (4) a rearrangement of surface molecules by surface diffusion. The theoretical framework for these mechanisms is given by Mullins^14,15^. He showed that for a one-dimensional sinusoidal indentation *u*_0_ sin (*kx*), the profile evolves as $U(x,t)=u\left(t\right){{\sin }}({kx})$, with *x* the direction perpendicular to the hole, *t* the time, and $u\left(t\right)$ the amplitude which is described by:

1 $$\frac{\partial u}{\partial t}=-\!{C}_{n}\left(T\right){k}^{n}u$$

where ${C}_{n}\left(T\right)$ is a temperature-dependent prefactor, ${k}$ the wavenumber of the profile, and *n* an integer that represents the physical mechanism behind the levelling:

- *n* = 1: a viscous-capillary flow, for an incompressible fluid and a low Reynolds number.
- *n* = 2: a dissolution-precipitation process, driven by the Kelvin equation that states that a curved surface has a higher saturation concentration and therefore can precipitate ions dissolved in its vicinity.
- *n* = 3: a bulk diffusive lattice flow, caused by interstitial or substitutional mechanisms in the bulk of the salt crystal.
- *n* = 4: surface diffusion, a rearrangement of surface molecules driven by the gradient of the curvature of the profile.

Since Mullins also showed that the sum of two solutions is again a solution, we decomposed the initial experimental profile at *t* = 0 as a Fourier sum and propagated each component individually in time (where we used a cut-off after the 90 lowest-spatial-frequency components). We found that the experimental data showed the best agreement with the first-order model describing the viscous flow process. This was obtained by a statistical test using chi-square minimization between the *u*-values of the data and the four candidate mechanisms.

Note that in the simplification of the model the small slope approximation was applied (*du/dx* ≪ 1) for both the projection of the molecular velocity and the curvature of the hole. The error bars are based on the pixel size in the raw data.

### Reporting summary

Further information on research design is available in the Nature Portfolio Reporting Summary linked to this article.

## Supplementary information

Supplementary Information

Description of Additional Supplementary Information

Supplementary Movie 1

Supplementary Movie 2

Reporting Summary

## Data Availability

Data are available for this paper on request. Correspondence and requests for materials should be addressed to N.S.

## Code availability

Codes are available for this paper on request. Correspondence and requests for materials should be addressed to N.S.
