# Author Correction: Softness of hydrated salt crystals under deliquescence

**DOI:** 10.1038/s41467-023-37124-5

**Published:** 2023-03-10

**Authors:** Rozeline Wijnhorst, Menno Demmenie, Etienne Jambon-Puillet, Freek Ariese, Daniel Bonn, Noushine Shahidzadeh

**Affiliations:** 1 Institute of Physics, Van der Waals-Zeeman Institute, University of Amsterdam, Science Park 904, 1098 XH Amsterdam, The Netherlands; 2 LaserLaB, Biophotonics and Medical Imaging, Vrije Universiteit Amsterdam, De Boelelaan 1081, 1081 HV Amsterdam, The Netherlands; 3 Present Address: Laboratory for Soft and Living Materials, Department of Materials, ETH Zurich, Vladimir-Prelog-Weg 1-5, Zurich, 8093 Switzerland

**Keywords:** Fluids, Chemical physics

Correction to: *Nature Communications* 10.1038/s41467-023-36834-0, published online 25 February 2023

The wrong Supplementary Movie 1 was originally published with this article; it has now been replaced with the correct. The original article has been corrected.

