# Supplementary material for: Softness of hydrated salt crystals under deliquescence
Source: Nat Commun. 2023 Feb 25;14:1090. doi: 10.1038/s41467-023-36834-0 (PMC9968288; doi:10.1038/s41467-023-36834-0)
Supplement: Supplementary file 1 — Supplementary Information [file 41467_2023_36834_MOESM1_ESM.pdf]

Softness of hydrated salt crystals under deliquescence

Rozeline Wijnhorst[1], Menno Demmenie[1], Etienne Jambon-Puillet[1]†, Freek Ariese[2], Daniel Bonn[1], Noushine Shahidzadeh[1]*

[1]Institute of Physics, Van der Waals-Zeeman Institute, University of Amsterdam, Science Park 904, 1098 XH Amsterdam, The Netherlands.

[2]LaserLaB, Biophotonics and Medical Imaging, Vrije Universiteit Amsterdam, De Boelelaan 1081, 1081 HV Amsterdam, The Netherlands.

†Current address: Laboratory for Soft and Living Materials, Department of Materials, ETH Zurich, Vladimir-Prelog-Weg 1-5, Zurich 8093, Switzerland.

*Corresponding author. Email: n.shahidzadeh@uva.nl.

Supplementary Information

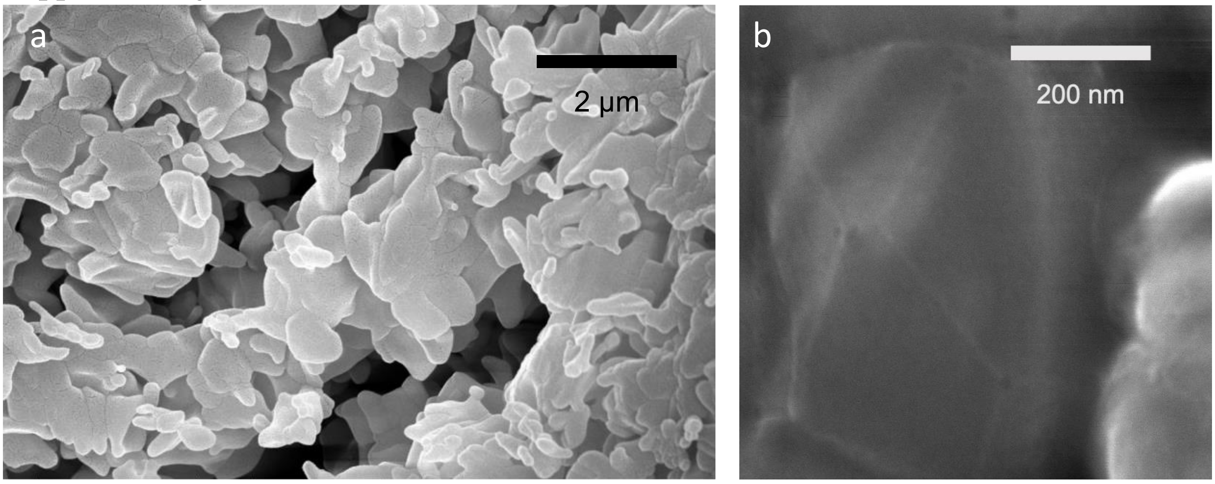

Supplementary Figure 1. **Electron microscopy images of a dried (dehydrated) mirabilite crystal composed of an assembly of thenardite nanocrystals. a** Microscale porous structure of the assembly **b** Facetted nanoscale structure of a single anhydrous sodium sulfate crystal (thernardite) from the assembly.

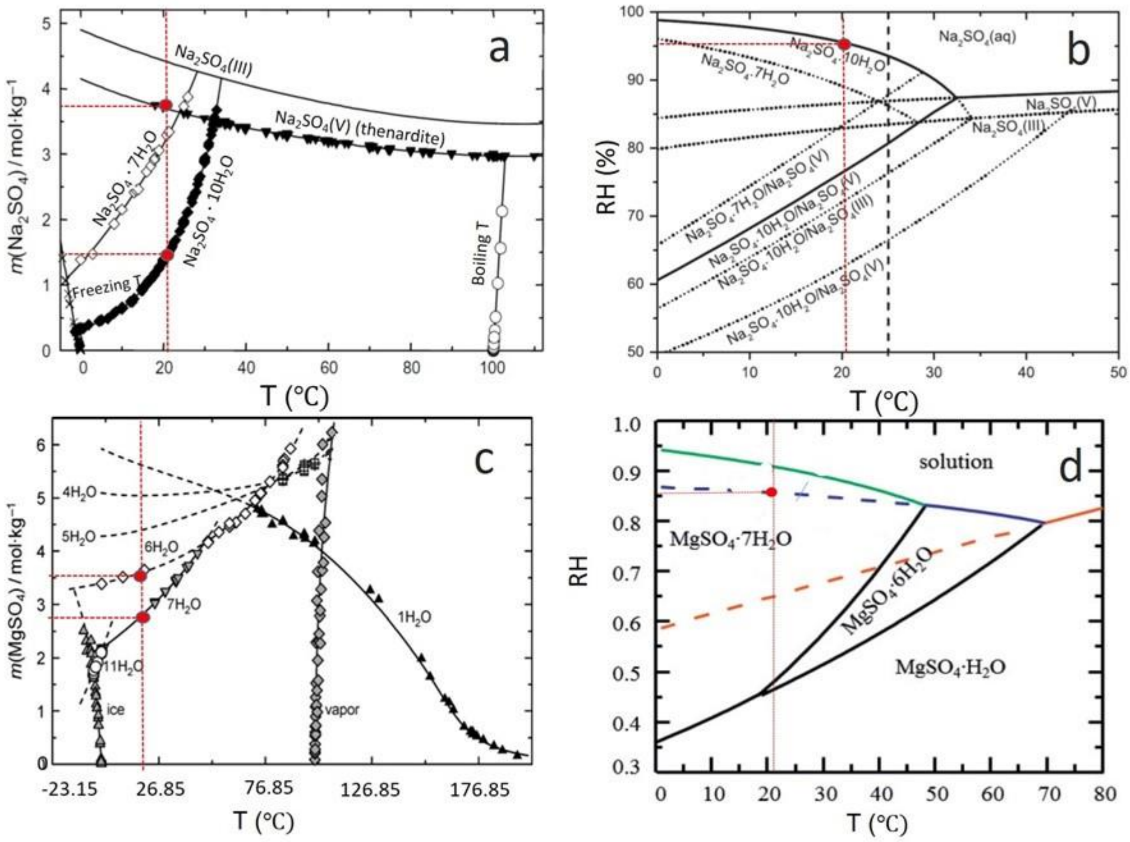

Supplementary Figure 2. **Phase diagrams of sodium sulfate and magnesium sulfate from the literature**. Solubility phase diagram **a** (Reprinted from Crystallization of sodium sulfate phases in porous materials: The phase diagram Na_2SO_4-H_2O and the generation of stress. *Geochim Cosmochim Acta* **72**, 4291–4306, Steiger, M. & Asmussen, S. Copyright (2008) with permission from Elsevier) **b** and humidity phase diagram *(*Reprinted from $Na_2SO4\cdot 10H_2O$ dehydration in view of thermal storage. *Chem Eng Sci* **134**, 360–366., Donkers, P. A. J., Linnow, K., Pel, L., Steiger, M. & Adan, O. C. G. Copyright (2015), with permission from Elsevier) of sodium sulfate mineral mirabilite (Na_2SO_4•$10H_2O$), heptahydrate (Na_2SO_4•$7H_2O$), and thenardite phase III and V (Na_2SO_4). **c** Solubility phase diagram (Reprinted from Publication Decomposition reactions of magnesium sulfate hydrates and phase equilibria in the $MgSO_4$-H_2O and Na^+-Mg^{2+}-Cl^--SO_4^{2-}-H_2O systems with implications for Mars., Steiger, M., Linnow, K., Ehrhardt, D. & Rohde, M. *Geochim Cosmochim Acta* **75**, 3600–3626 Copyright(2011), with permission from Elsevier) **d** and humidity phase diagram (Reprinted from Experimental studies of the mechanism and kinetics of hydration reactions., Linnow, K., Niermann, M., Bonatz, D., Posern, K. & Steiger, M. in *Energy Procedia* vol. 48 394–404 Copyright (2014), with permission from Elsevier) of magnesium sulfate epsomite ($MgSO_4$•$7H_2O$), hexahydrate ($MgSO_4$•$6H_2O$), and kieserite ($MgSO_4$•H_2O). The red dotted lines represent our experimental conditions. Generally, the higher the number of water molecules in the crystalline structure of an inorganic hydrated crystal, the lower its solubility at a given temperature[1–4]

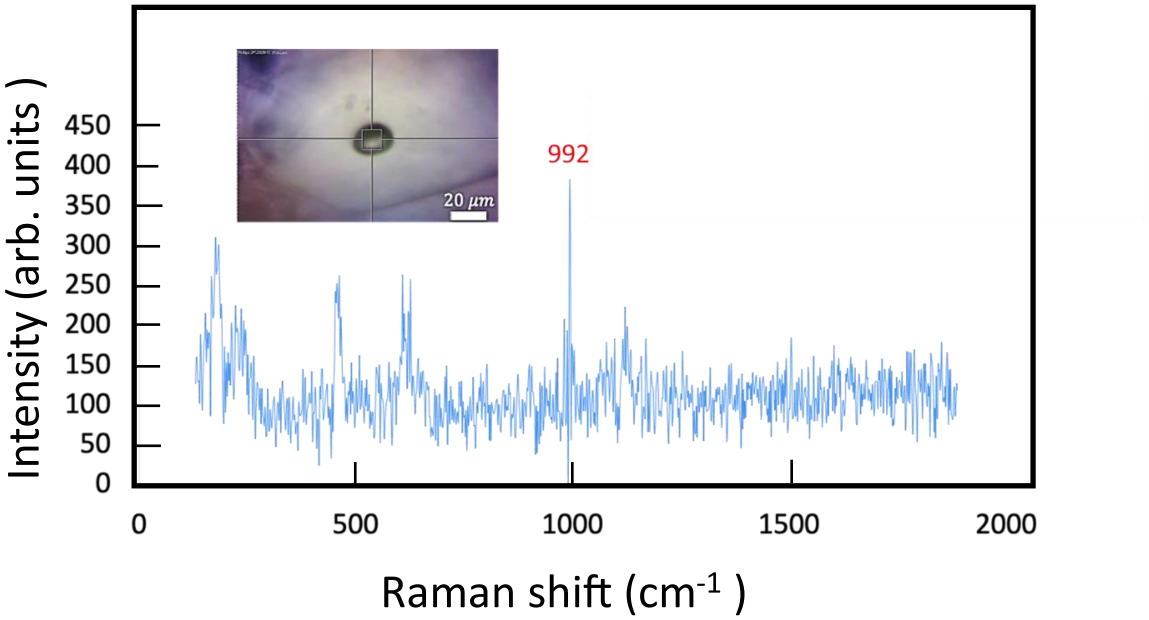

Supplementary Figure 3. **Subtracted Raman spectrum of the ‘black spots’ and the mirabilite crystal surrounding it.** The peak at 992±2 cm^{-1} corresponds to 1.3% thenardite, the anhydrous form of sodium sulfate.

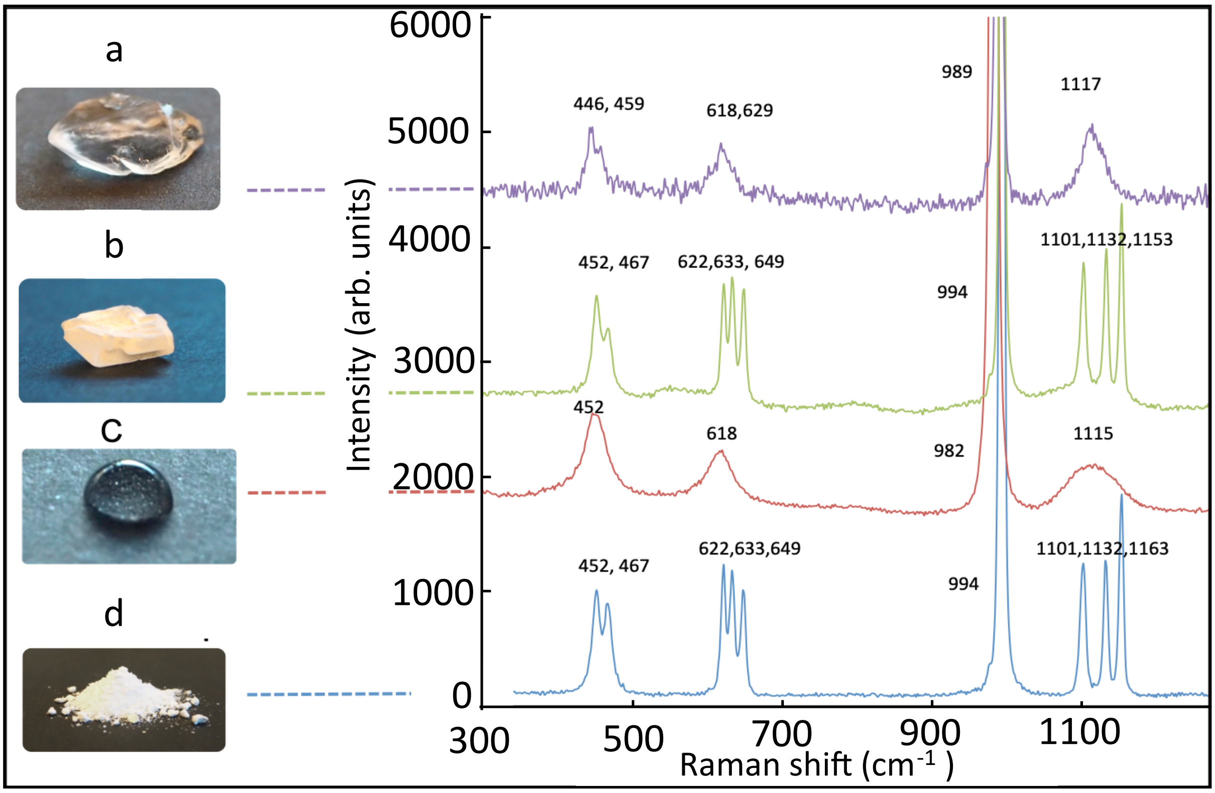

Supplementary Figure 4. **Raman spectra of different phases of sodium sulfate. a** Mirabilite crystal (Na_2SO_4•$10H_2O$). **b** Crystal of image a Dried at room temperature, identified as thenardite (anhydrous Na_2SO_4). **c** Aqueous Na_2SO_4 solution. **d** Crushed crystal, (image b) identified as thenardite (anhydrous Na_2SO_4). Spectra are vertically offset for clarity; intensities in arbitrary units.

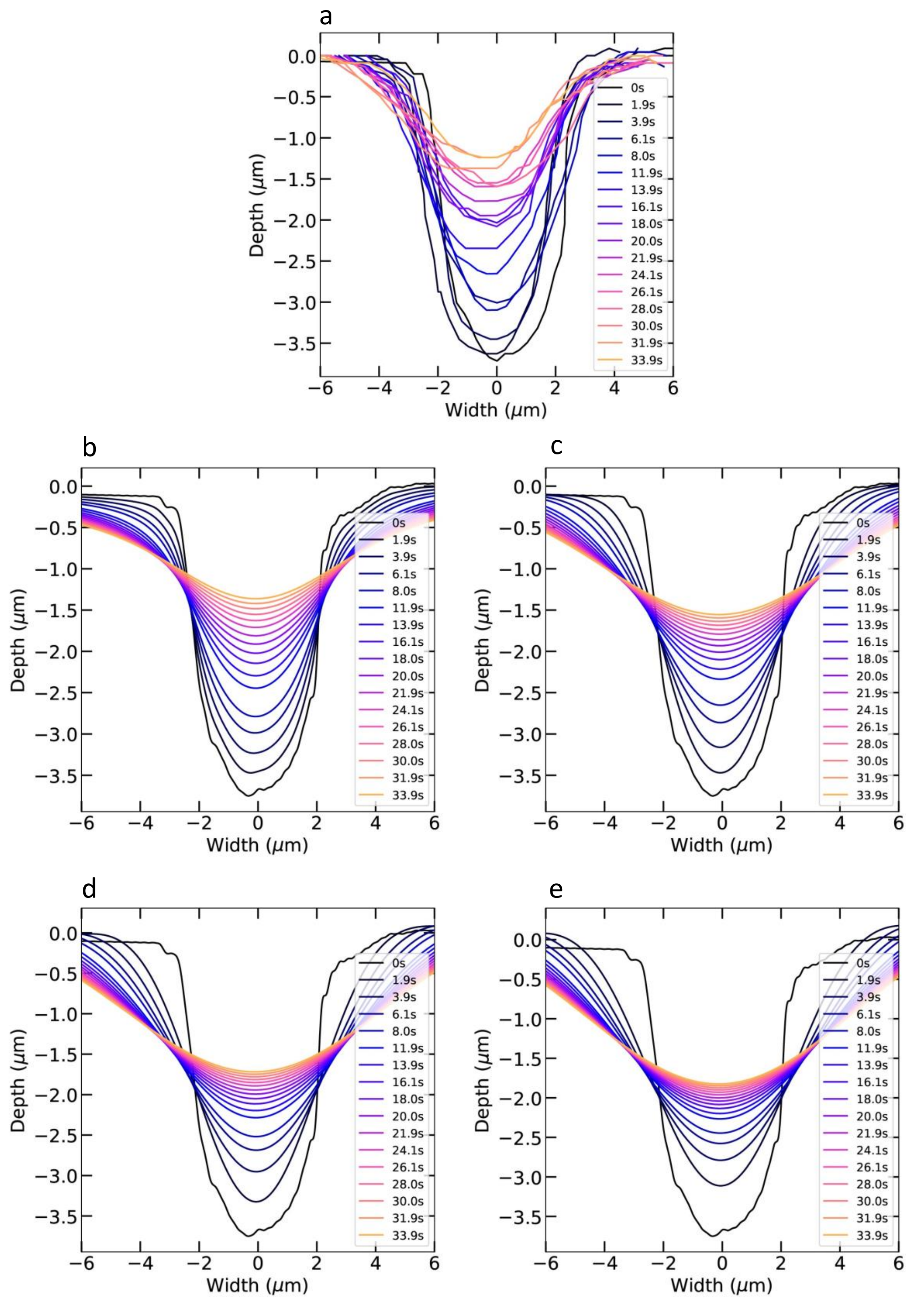

Supplementary Figure 5. **Comparison of the four models with mirabilite self-healing data.** **a** Raw data. **b** Effective flow model (n = 1). **c** Dissolution precipitation model (n = 2). **d** Bulk lattice flow model (n = 3). **e** Rearrangement of surface molecules by surface diffusion model (n = 4).

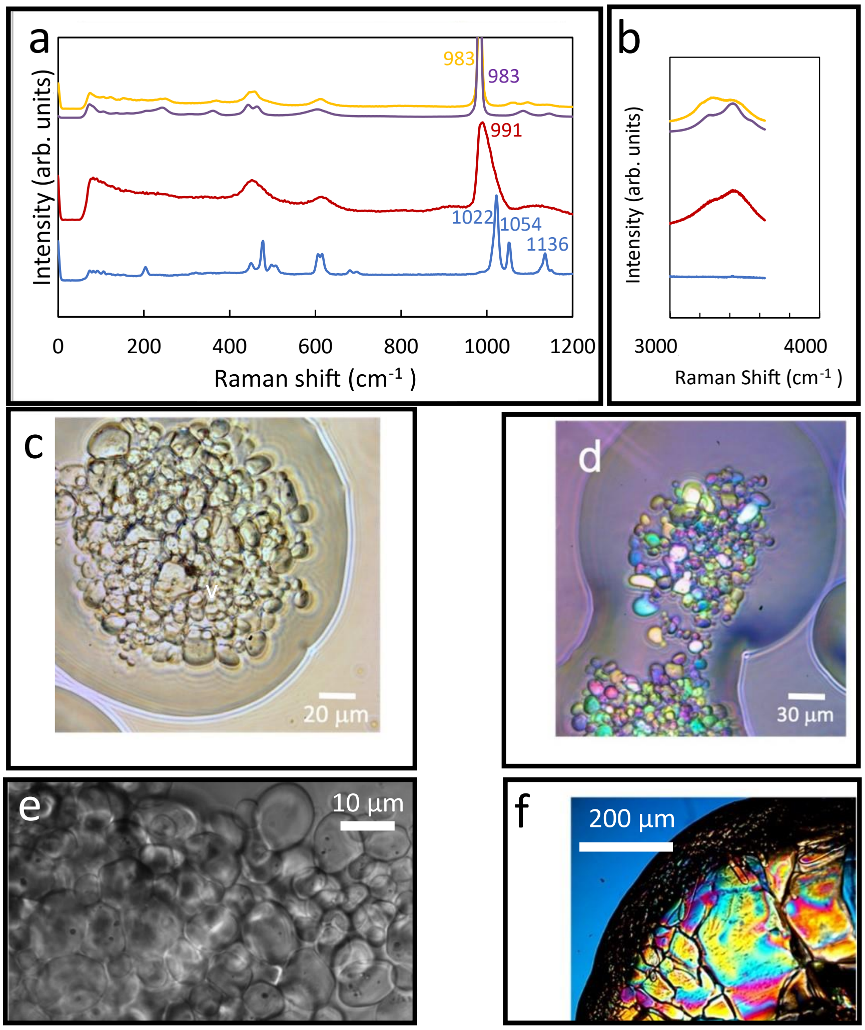

Supplementary Figure 6. **Raman and optical microscopy of magnesium sulfate. a, b** Raman spectrum of a ‘deformable’ magnesium sulfate crystal (peak at 983 cm^{-1}, yellow line) compared to that of the hexahydrate crystal (983 cm^{-1}, purple), a saturated solution of magnesium sulfate (991 cm^{-1}, red), and anhydrous magnesium sulfate powder (1022, 1054, and 1136 cm^{-1}, blue). **c-e** Deformable magnesium sulfate crystals under optical and polarizing light microscope formed during deliquescence. **f** Hexahydrate of magnesium sulfate in a dried droplet under the polarizing light microscope.

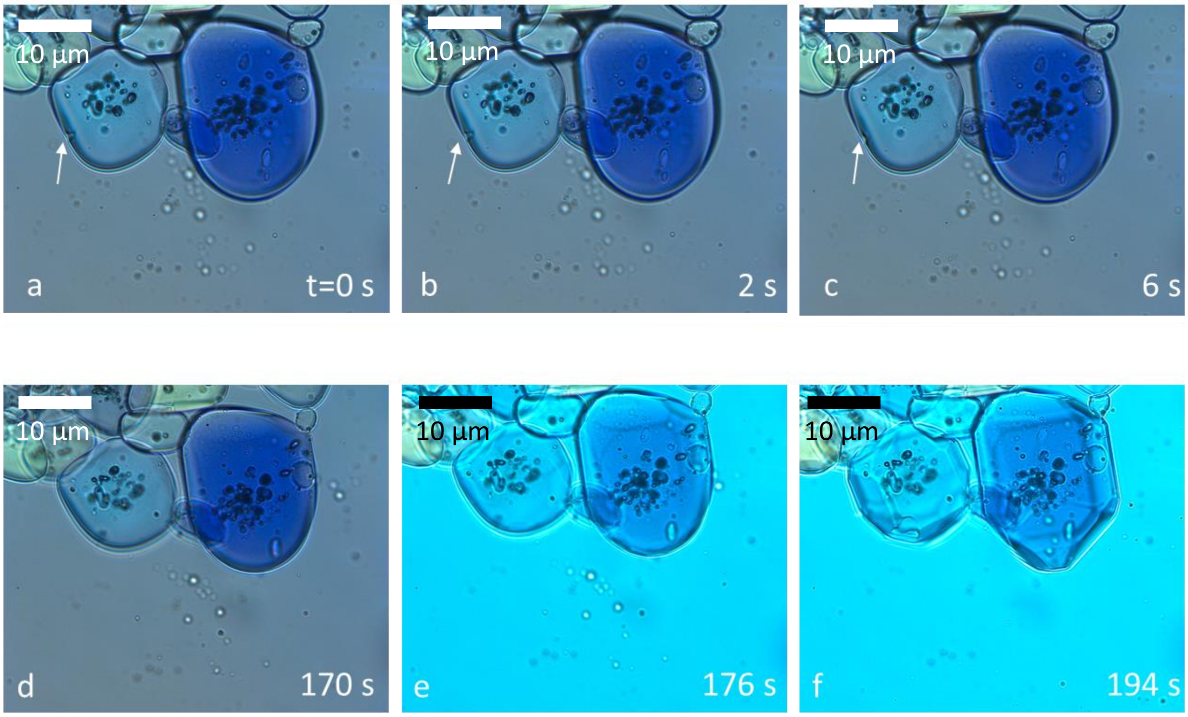

Supplementary Figure 7. **Behavior of mirabilite crystals below and above the equilibrium relative humidity (RH_{eq}=96%). a-d** Deliquescence at RH ~100%, T=21ºC. The crystal is soft and a defect (indicated by the arrow) levels off rapidly. **e-f** Crystal growth and facetting at RH~60%, T=21ºC, after removal from the deliquescence chamber. The crystal evolves towards a well facetted and rigid surface.

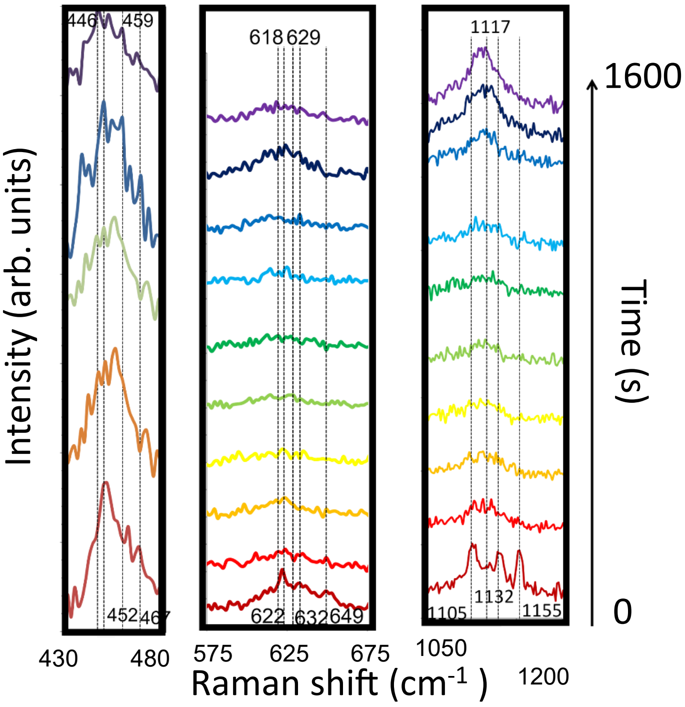

Supplementary Figure 8**. Raman spectra during the gradual deliquescence of thenardite (Na_2SO_4).**

Supplementary Table 1 | Levelling velocity of holes in mirabilite crystal walls for holes of different depths. The velocity value is determined by the best fitting value $C_1(T)$ of the first order model.

Hole depth (μm)	Levelling velocity (μm^{-1})
4.2	0.205 ± 0.03
1.8	0.096 ± 0.03
4.2	0.535 ± 0.08
12.1	0.143 ± 0.01

Supplementary References

1. Steiger, M., Linnow, K., Ehrhardt, D. & Rohde, M. Decomposition reactions of magnesium sulfate hydrates and phase equilibria in the MgSO4-H2O and Na+-Mg2+-Cl--SO42--H2O systems with implications for Mars. *Geochim Cosmochim Acta* **75**, 3600–3626 (2011).
2. Steiger, M. & Asmussen, S. Crystallization of sodium sulfate phases in porous materials: The phase diagram Na2SO4-H2O and the generation of stress. *Geochim Cosmochim Acta* **72**, 4291–4306 (2008).
3. Donkers, P. A. J., Linnow, K., Pel, L., Steiger, M. & Adan, O. C. G. Na2SO4·10H2O dehydration in view of thermal storage. *Chem Eng Sci* **134**, 360–366 (2015).
4. Linnow, K., Niermann, M., Bonatz, D., Posern, K. & Steiger, M. Experimental studies of the mechanism and kinetics of hydration reactions. in *Energy Procedia* vol. 48 394–404 (Elsevier Ltd, 2014).
