# Supplementary material for: Softness of hydrated salt crystals under deliquescence
Source: Nat Commun. 2023 Feb 25;14:1090. doi: 10.1038/s41467-023-36834-0 (PMC9968288; doi:10.1038/s41467-023-36834-0)
Supplement: Supplementary file 2 — Description of Additional Supplementary Information [file 41467_2023_36834_MOESM2_ESM.pdf]

Description of Additional Supplementary Files

Title: Supplementary movie 1

Description: Videos of deliquescence of anhydrous sodium sulfate to the deformable state of mirabilite

Title: Supplementary Movie 2

Description: Deliquescence of NaCl
